# Antiproliferative Effect of Elastin-Derived Peptide VGVAPG on SH-SY5Y Neuroblastoma Cells

**DOI:** 10.1007/s12640-019-00040-y

**Published:** 2019-06-03

**Authors:** Konrad A. Szychowski, Agnieszka Rombel-Bryzek, Agnieszka Dołhańczuk-Śródka, Jan Gmiński

**Affiliations:** 1 Department of Clinical Biochemistry and Laboratory Diagnostics, Institute of Medicine, University of Opole, Oleska 48, 45-052 Opole, Poland; 2 Institute of Biotechnology, University of Opole, Kard. B. Kominka 6a, 45-032 Opole, Poland; 3 Department of Public Health, Dietetics and Lifestyle Disorders, Faculty of Medicine, University of Information Technology and Management in Rzeszow, Sucharskiego 2, 35-225 Rzeszow, Poland

**Keywords:** Elastin-derived peptides, VGVAPG, ROS, SH-SY5Y, Proliferation

## Abstract

Throughout the lifetime of humans, the amount of stem cells and the rate of cell proliferation continue to decrease. Reactive oxygen species (ROS) are one among the many factors that promote stem cell aging. Both a decrease in the level of stem cells and increase in ROS production can lead to the development of different neurodegenerative diseases. This study was conducted to determine how the VGVAPG peptide, liberated from elastin during the aging process and under pathological conditions, affects ROS production and activities of antioxidant enzymes in undifferentiated, proliferating SH-SY5Y cells. SH-SY5Y cells were maintained in Dulbecco’s modified Eagle’s medium/nutrient mixture F-12 supplemented with 10% heat-inactivated fetal bovine serum (FBS). After treating the SH-SY5Y cells with VGVAPG peptide, we measured ROS production; cell metabolism, proliferation, and expression; and activities of superoxide dismutase (SOD), glutathione peroxidase (GPx), and catalase (CAT). We demonstrated that the VGVAPG peptide increases GPx expression and activity, whereas it decreases CAT expression in SH-SY5Y cells. Silencing of the *GLB1* gene prevents changes in GPx activity. Despite the fact that the VGVAPG peptide increases GPx expression, it increases the ROS level. Moreover, the VGVAPG peptide decreases SH-SY5Y proliferation, which is prevented by the ROS scavenger *N*-acetyl-L-cysteine. Our data suggest that ROS production and decreased proliferation of SH-SY5Y cells are the results of excitotoxicity meditated through close unrecognized molecular pathways. More research is needed to elucidate the unknown mechanism of action of VGVAPG peptide in the nervous system.

## Introduction

Throughout the human lifetime, the amount of stem cells and rate of cell proliferation continue to decrease (Apple et al. 2017). The presence of reactive oxygen species (ROS) is one among the many factors that promote stem cell aging (Oh et al. 2014). To date, it has been described that the decrease in stem cell level and increase in ROS production can both lead to the development of different neurodegenerative diseases (Kim et al. 2015). During the aging process and with exposure to xenobiotics, there is a consequent increase in ROS levels in the brain that then generates a snowball phenomenon (Kołodziej et al. 2018). Moreover, ROS have been suggested to be involved in the evolution of cellular damage caused by ischemia–reperfusion, anoxia–reperfusion, and hypoxia–reperfusion (Sasaki et al. 2018). ROS damages the cells and walls of brain capillaries, and this leads to the release of elastin-derived peptides (EDPs) from the extracellular matrix (ECM) (Nita and Grzybowski 2016). In the elastin molecule, the VGVAPG peptide is the most important sequence—one that is repeated many times and is easily liberated from either elastin or EDPs under physiological and pathological conditions (Senior et al. 1984; Gminski et al. 1993). It is well documented that the VGVAPG sequence (alone or as a part of EDPs) binds with high affinity to elastin-binding protein (EBP) located on the cell surface (Senior et al. 1984; Blood et al. 1988). EBP is a catalytically inactive form of the alternatively spliced gene for β-galactosidase (*GLB1* gene) (Hinek et al. 1993; Skeie et al. 2012). The second receptor for the VGVAPG peptide is galectin-3, which also has an important role in cell–ECM interactions (Ochieng et al. 2002). Galectin-3 is mostly expressed in inflammatory cells (Bresalier et al. 1996; Cantarelli et al. 2009); however, its expression has been linked with tumor progression, cancer aggressiveness, and melanoma invasiveness (Ochieng et al. 1999; Pocza et al. 2008; Wang et al. 2009).

Studies, to date, have reported that EDPs induce ROS production in monocytes and human fibroblasts (Robert et al. 1984; Scandolera et al. 2015). Our previous research showed that ROS levels increase during stimulation with VGVAPG peptide in mouse primary astrocytes in vitro (Szychowski and Gmiński 2019a). It has been described that EDPs enhance the activities of antioxidant enzymes, such as superoxide dismutase (SOD), catalase (CAT), or glutathione peroxidase (GPx), and increase lipid peroxidation in human fibroblasts (Gmiński et al. 1991). Moreover, the VGVAPG peptide reduced ROS production in neutrophils in control patients and those with stable chronic obstructive pulmonary disease (COPD) (Dupont et al. 2013). Interestingly, at low concentrations, ROS can act as signaling molecules in both intra- and extracellular signal transduction pathways to influence a variety of cellular processes, such as proliferation, metabolism, differentiation, and survival (Glennon-Alty et al. 2018).

The involvement of the VGVAPG peptide and EDPs in cell proliferation is well documented in human skin fibroblasts, in the cytotrophoblast in first-trimester placental explants, in astrocytoma and glioblastoma cell lines, as well as in porcine coronary arterial smooth muscle cells (Kamoun et al. 1995; Jung et al. 1998; Mochizuki et al. 2002; Coquerel et al. 2009; Desforges et al. 2014). Cell proliferation is mainly associated with the activation of EBP and, less often, with galectin-3, which is mainly involved in cell migration (Inohara et al. 1998; Toupance et al. 2012).

Human neuroblastoma (SH-SY5Y) cells maintain their potential for proliferation and differentiation under culture conditions and display some properties of stem cells (Walton et al. 2004; Hämmerle et al. 2013; Ross et al. 2015). Therefore, because of their stemness, SH-SY5Y cells can be used as a model of undifferentiated neuroblasts to test cell proliferation (Walton et al. 2004; Hämmerle et al. 2013). Recent data have shown that SH-SY5Y cells are a good model for testing ROS-dependent apoptosis and cell proliferation in neurological conditions such as Alzheimer’s and Parkinson’s disease (Uğuz et al. 2016; Venkatesh Gobi et al. 2018). Moreover; an increase in ROS production in the SH-SY5Y cell line can be associated with autophagy, which is present in neurological diseases (Chiappini et al. 2018).

This study was conducted with an aim to determine how the VGVAPG peptide affects ROS production as well as the expression and activities of antioxidant enzymes in undifferentiated, proliferating SH-SY5Y cells.

## Materials and Methods

### Reagents

DMEM/F12 1:1 (16–405-CVR) without phenol red was purchased from Corning (Manassas, VA, USA). Trypsin, streptomycin, penicillin, glycerol, CHAPS, HEPES, dithiothreitol (DTT), NaCl, EDTA, dimethyl sulfoxide (DMSO), 2′,7′-dichlorodihydrofluorescein diacetate (H_2_DCFDA), and *N*-acetyl-L-cysteine (NAC) were purchased from Sigma-Aldrich (St. Louis, MO, USA). The FastStart Universal Probe Master (Rox) was purchased from Roche Applied Science (Mannheim, Germany). The *GLB1* siRNA (sc-43792) and *LGALS3* (sc-155994) were purchased from Santa Cruz Biotechnology (Santa Cruz, CA, USA). The VGVAPG and VVGPGA peptides were synthesized by LipoPharm.pl (Gdańsk, Poland). Heat-inactivated fetal bovine serum (FBS) was purchased from EURx (Gdańsk, Poland). The High-Capacity cDNA Reverse Transcription Kit and the TaqMan® probes corresponding to specific genes encoding *ACTB* (Hs01060665_g1), *PPARγ* (Hs00234592_m1), and *KI67* (Hs04260396_g1) were obtained from Life Technologies Applied Biosystems (Foster City, CA, USA). Peroxisome proliferator-activated receptor gamma (PPARγ; E-EL-H1361), SOD1 (E-EL-H1113), and GPx (E-EL-H5410) ELISA assays as well as CAT (E-BC-K031), SOD (E-BC-K020), and GPx (E-BC-K096) activity assays were obtained from Elabscience Biotechnology (Wuhan, China). CAT (EH0643) and Ki67 (EH0684) ELISA assays were obtained from Wuhan Fine Biotech Co., Ltd. (Wuhan, China). Stock solutions of the VGVAPG and VVGPGA peptides were prepared in DMSO and added to the DMEM/F12 medium. The final concentration of DMSO in the culture medium was always 0.1%.

### SH-SY5Y Cell Culture

The human neuroblastoma (SH-SY5Y) cell line were obtained from the American Type Culture Collection (ATCC distributor: LGC Standards, Łomianki, Poland). SH-SY5Y cells were maintained in DMEM/F12 medium supplemented with 10% heat-inactivated FBS at 37 °C in a humidified atmosphere with 5% CO_2_. The cells were seeded in 96-well culture plates at a density of 6 × 10^3^ (for 6- and 24-h treatment) and 5 × 10^3^ (for 48-h treatment) per well, and initially cultured for 24 h prior to the experiment. Subsequently, the medium was changed to a fresh medium by increasing the concentrations (100 pM; 1, 10, 50, and 100 nM; and 1, 10, 50, and 100 μM) of the VGVAPG peptide.

### siRNA Gene Silencing Procedure

*GLB1* and *LGALS3* siRNA were used to inhibit the expression of EBP and galectin-3 in the SH-SY5Y cell line. The experiment was conducted in accordance with a previously described procedure (Szychowski et al. 2019a). Briefly, *GLB1* and *LGALS3* siRNA were applied for 7 h at a final concentration of 50 nM in an antibiotic-free medium containing the siRNA transfection reagent INTERFERin. After transfection, the culture medium was changed to normal culture medium and the SH-SY5Y cells were cultured for 12 h prior to commencing the experiment. Vehicle controls included positive siRNA and scramble siRNA containing a scrambled sequence that did not lead to specific degradation of any known cellular mRNA.

### Measurement of ROS Production

The ROS measurement was undertaken according to a previously described method (Szychowski and Wójtowicz 2016). To determine the ability of the VGVAPG peptide to induce ROS production in the selected cells, 5 μM H_2_DCFDA was applied. The cells were then incubated in H_2_DCFDA in serum-free and phenol red–free medium for 45 min prior to treatment with compounds. After 3-, 6-, and 24-h incubation of the cells with increasing concentrations of compounds (5% CO_2_ at 37 °C), the culture medium was replaced with fresh medium to remove the extracellular residual DCF and the studied compound to reduce the background fluorescence. Cells treated with 0.3% hydrogen peroxide (H_2_O_2_) were used as a positive control (result not shown). DCF fluorescence was measured using a microplate reader (FilterMax F5) at maximum excitation and emission spectra of 485 and 535 nm, respectively.

### Resazurin Reduction Assay

The resazurin reduction cell viability and metabolism assay was conducted according to a previously described method (Szychowski et al. 2017a). On the day of analysis, a working solution of 60 μM resazurin was prepared in a medium containing 1% FBS. After 24- and 48-h treatment, cells with increasing concentrations of the VGVAPG peptide medium in the wells were replaced into a working solution of resazurin (100 μL) and the plates were placed in 37 °C. Fluorescence was measured with excitation and emission wavelengths of 530 and 590 nm, respectively, on a FilterMax F5 Multi-Mode microplate reader (Molecular Devices, Corp., Sunnyvale, CA, USA) for 30 min and 1 h after dye addition.

### Real-Time PCR Analysis of mRNAs Specific to Genes Encoding *Ki67* and *PPARγ*

For the real-time PCR assay, the SH-SY5Y cell line was seeded on six-well plates and initially cultured for 24 h. After 6-h exposure to 50 nM and 1 μM of VGVAPG, samples of total RNA were extracted from the SH-SY5Y cell line in accordance with the manufacturer’s protocol, based on a previously described method (Szychowski et al. 2017b). After siRNA transfection, the procedure was undertaken after exposure to a concentration of 50 nM and 1 μM VGVAPG for 6 h. The RNA quality and quantity were determined spectrophotometrically at 260 and 280 nm, respectively (ND/1000 UV/Vis; Thermo Fisher NanoDrop, USA). Two-step quantitative real-time reverse transcription (qRT) PCR was conducted using the CFX Real-Time System (BioRad, USA). The RT reaction was carried out at a final volume of 20 μL with 180 ng of RNA (as a cDNA template) by using the cDNA reverse transcription kit according to the manufacturer’s instructions. Briefly, the reaction was conducted for 10 min at 25 °C, then 120 min at 37 °C, next for 5 min at 85 °C, and thereafter held at 4 °C. Between analyses, cDNA was stored at − 80 °C. Products from the RT reaction were amplified using the FastStart Universal Probe Master (Rox) kit with TaqMan probes as primers for specific genes encoding *ACTB*, *PPARγ*, and *KI67* according to the manufacturer’s protocol. Amplification was carried out in a total reaction volume of 20 μL containing 1.0 μL RT product; *ACTB* was used as the reference gene. A standard qPCR procedure was utilized: the reaction was conducted for 2 min at 50 °C and 10 min at 95 °C, followed by 40 cycles of 15 s at 95 °C and 1 min at 60 °C. Random peptide sequence VVGPGA was used as a control, and did not affect *PPARγ* and *KI67* mRNA expression (data not shown). The threshold value (Ct) for each sample was set during the exponential phase, and the ΔCt method was used for data analysis. To study the gene expression levels, three candidate reference genes (*Actb*, *Gapdh*, and *18S*) were selected and validated. To evaluate the reference gene expression, we used the RefFinder web-based comprehensive tool, which integrates major computational programs (geNorm, Normfinder, BestKeeper, and the comparative ΔCt method) for comparison and ranking of candidate reference genes.

### ELISA for Ki67, PPARγ, SOD1, CAT, and GPx

Levels of Ki67, PPARγ, SOD1, CAT, and GPx proteins were determined via ELISA after 24- and 48-h treatment with 50 nM and 1 μM VGVAPG. Specific detections of these proteins were obtained using ELISA and they were subsequently subjected to quantitative sandwich enzyme immunoassay. The assay was conducted according to the manufacturer’s instructions from Elabscience Biotechnology and Wuhan Fine Biotech Co., Ltd. (Wuhan, China). Briefly, a 96-well plate was pre-coated with monoclonal antibodies specific for Ki67, SOD1, CAT, and GPx. Standard references and the collected cell extracts were added to the wells and incubated for 90 min at 37 °C. Then, after the supernatant was removed, 100 μL biotinylated detection antibody was added for 60 min. After washing three times to remove any unbound substances, horseradish peroxidase-conjugated avidin was added to the wells. Following additional washing, 90 μL substrate solution was added to the wells for 15 min. Then, 50 μL reaction termination solution was added, and absorbance was measured at 450 nm using a microplate reader (FilterMax F5); this value was proportional to the amount of Ki67, SOD1, CAT, and GPx. The protein concentration was measured in each sample and determined in triplicate for each sample by using the Thermo Fisher NanoDrop device.

### Enzyme Activity Assays

In all activity assays, the protein concentration was spectrophotometrically determined in triplicate for each sample at 280 nm by using the ND/1000 UV/Vis, Thermo Fisher NanoDrop device.

#### GPx Activity Assay

The activity of the GPx enzyme was assessed according to the manufacturer’s protocol. All samples were prepared in either non-enzyme or enzyme tubes. The enzymatic reaction was prepared according to the schema: 0.2 mL of 1 mM GSH and 0.1 mL of reagent 1 were added to the enzyme tube. After incubation for 5 min in a water bath at 37 °C, 2 mL of reagent 2 was added. In the non-enzymatic tube, the sample was added after the addition of reagent 2. Following the addition of all components, the tubes were centrifuged at 3100×*g* for 10 min. After centrifugation, we added 1, 0.25, and 0.05 mL of reagents 3, 4, and 5, respectively, to 1 mL of supernatant. All the reagents were mixed and incubated for 15 min at room temperature. The OD values for each tube were measured at 412 nm using a microplate reader (ELISA LEDETECT 96).

#### SOD Activity Assay

The SOD activity was assessed according to the manufacturer’s protocol. To the control well, we added 20 μL distilled water and 20 μL enzyme working solution. To the control-blank well, we added 20 μL distilled water and 20 μL enzyme diluent. To the sample well, we added 20 μL sample and 20 μL enzyme working solution. To the sample-blank well, we added 20 μL sample and 20 μL enzyme diluent. Thereafter, 200 μL substrate solution was added to each well and the 96-well microplate was incubated at 37 °C for 20 min. The OD values were read at 450 nm using a microplate reader (ELISA LEDETECT 96).

#### CAT Activity Assay

The activity of the CAT enzyme was assessed according to the manufacturer’s protocol. To the sample tube, we added 10 μL sample, 100 μL reagent 1, and 10 μL reagent 2, whereas we added only reagents 1 and 2 in the control tube. The tubes were shaken and incubated at 37 °C for 1 min. After incubation, reagent 3 (100 μL) and reagent 4 (10 μL) were added to the sample and control tubes, respectively; moreover, 10 μL sample was added to the control tube. The reagents were mixed and allowed to stand for 10 min at room temperature. The OD value of each tube (230 μL) was measured in a 96-well microplate at 405 nm using a microplate reader (ELISA LEDETECT 96).

### Statistical Analysis

Data are presented as the mean ± standard deviation (SD) of three independent experiments. In each experiment, the treatment was repeated six times (*n* = 6). Data are presented as a percentage of the control and analyzed via one-way analysis of variance (ANOVA) followed by Tukey’s multiple comparison procedure (*p* denotes the probability value; ***p < 0.001, **p < 0.01, and *p < 0.05).

## Results

### ROS Production

**Fig. 1:**
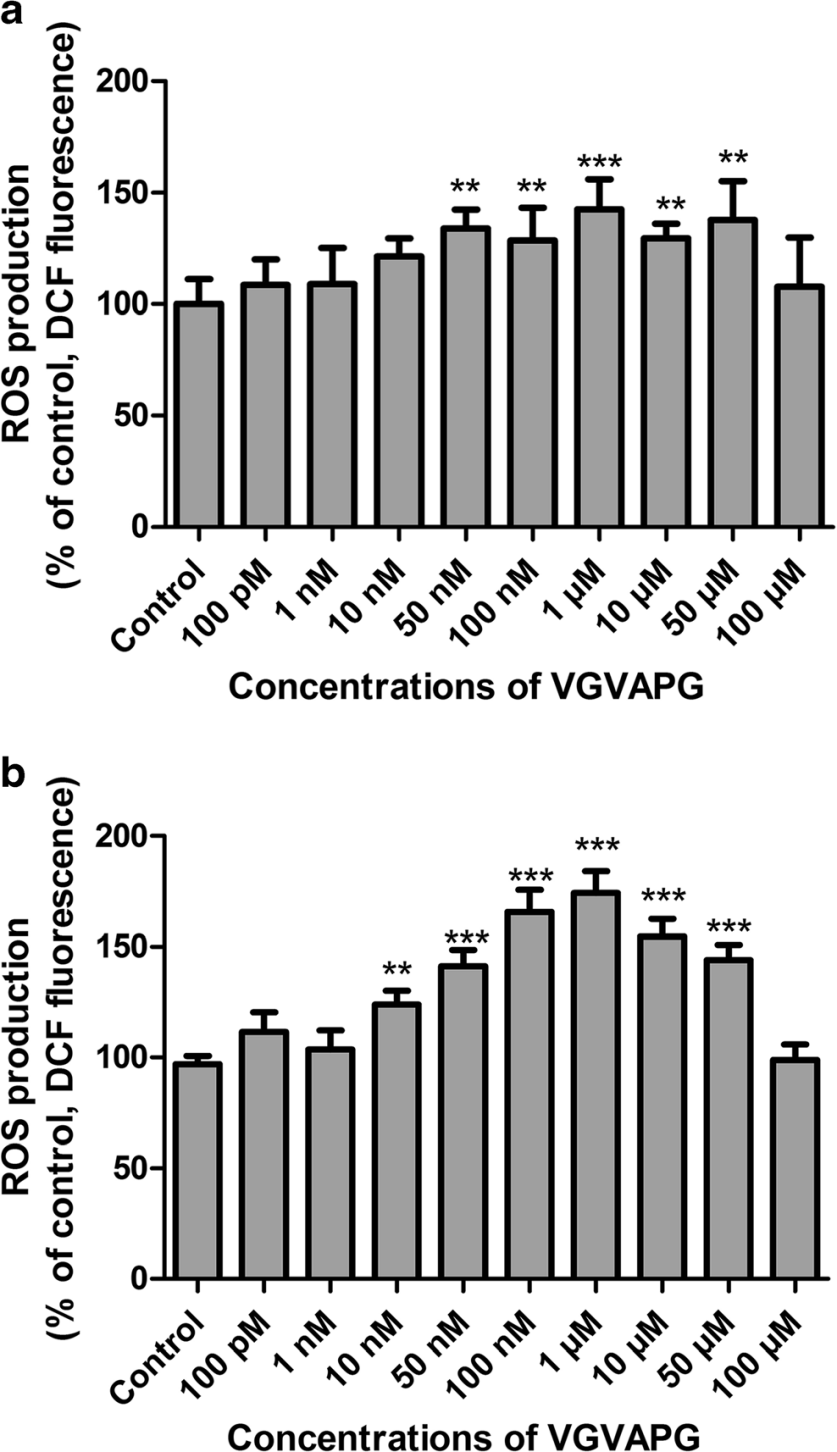
Effect of different (100 pM to 100 μM) VGVAPG peptide concentrations on DCF fluorescence in the SH-SY5Y cell line. After **a** 3 h of exposure and **b** 6 h of exposure. Each point represents the mean ± SD of three independent experiments, each of which consisted of six replicates per treatment group. **p < 0.01, ***p < 0.001, versus the control cells

After the SH-SY5Y cells were exposed to concentration of 100 pM to 100 μM VGVAPG for 3 h only in the range 50 nM to 50 μM, respectively, samples with the VGVAPG concentration showed increased ROS production by 33.98 to 37.82% compared to the control (Fig. 1a). Similarly, ROS production increased by 44.45 to 47.25% compared to the control (Fig. 1b) after SH-SY5Y cells were exposed for 6 h to concentrations of 10 nM to 50 μM VGVAPG.

On 24-h exposure of SH-SY5Y cells to 1 μM VGVAPG and 1 μM VVGPGA, respectively, only VGVAPG was shown to increase ROS production by 20.22% as compared to the control. The NAC as a ROS scavenger did not affect ROS production as compared to the control; however, NAC as co-treatment with VGVAPG prevented ROS production (ROS production remained unchanged as compared to the control; Fig. 3a).

### Resazurin Reduction Assay

**Fig. 2:**
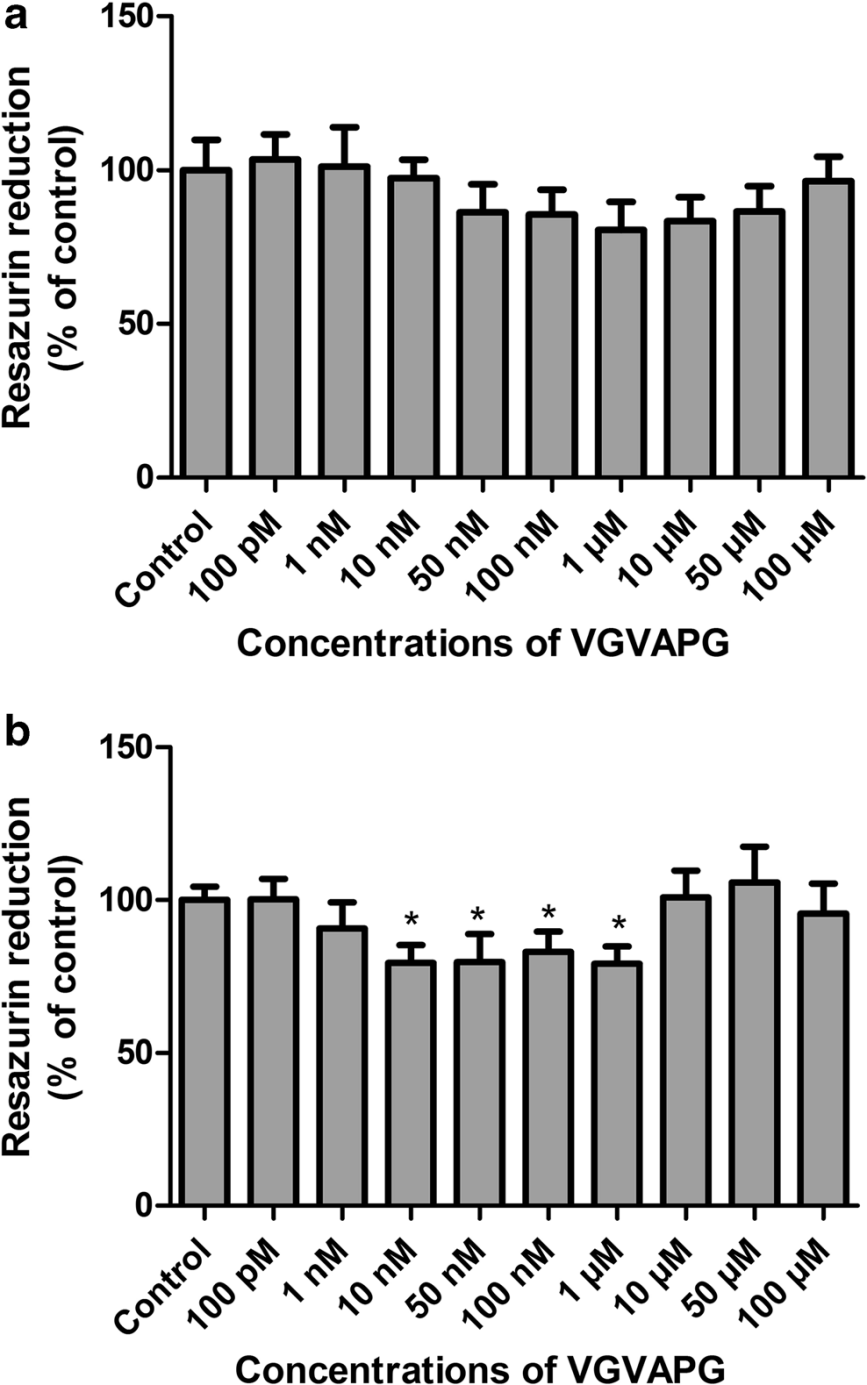
The effect of different (100 pM to 100 μM) VGVAPG peptide concentrations on resazurin reduction in the SH-SY5Y cell line. After **a** 24 h of exposure and **b** 48 h of exposure. Each point represents the mean ± SD of three independent experiments, each of which consisted of six replicates per treatment group. *p < 0.05, versus the control cells

No statistically significant decrease in resazurin reduction was observed as compared to the control after 24-h exposure of SH-SY5Y cells to concentrations of 100 pM to 100 μM VGVAPG; nevertheless, a downward trend was visible (Fig. 2a). Following 48-h exposure of SH-SY5Y cells to concentrations of 100 pM to 100 μM VGVAPG, we observed that 10 nM to 1 μM concentrations decreased resazurin reduction by 20.51 to 20.76% as compared to control (Fig. 2b).

**Fig. 3:**
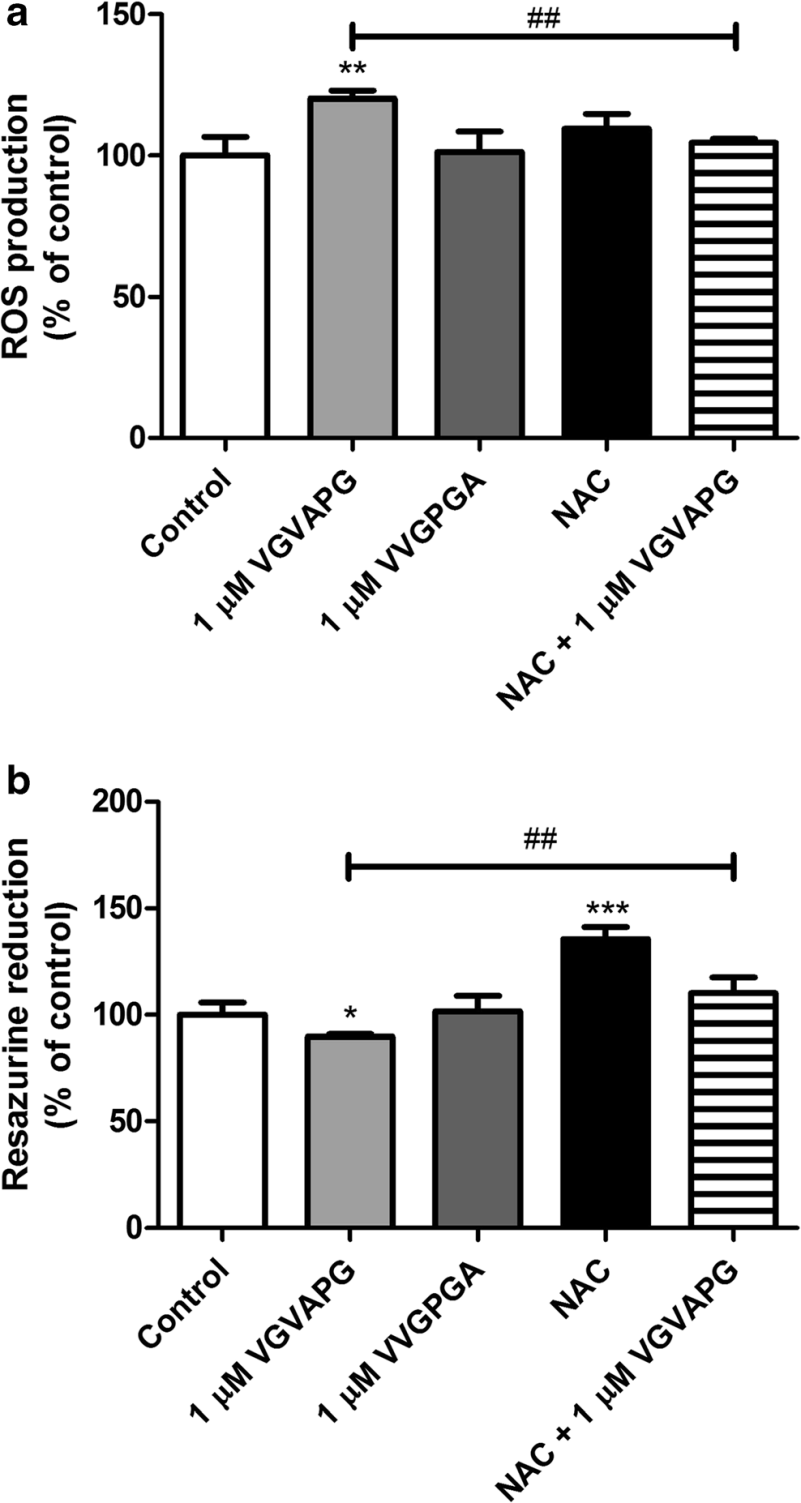
**a** The effect of 1 μM VGVAPG peptide on ROS production in the SH-SY5Y cell line after 24 h. **b** The effect of 1 μM VGVAPG peptide on resazurin reduction in the SH-SY5Y cell line after 48 h. White bars represent control cells, gray bars show cells treated with 1 μM VGVAPG, black bars represent cells treated with NAC, and bars with stripes indicate cells co-treated with 1 μM VGVAPG and 1 μM NAC. Every point represents the mean ± SD of three independent experiments, each of which comprises six replicates per treatment group. *p < 0.05, **p < 0.01, ***p < 0.001, versus the control cultures. ##*p* < 0.01, VGVAPG versus VGVAPG with NAC

After 24-h exposure of SH-SY5Y cells to 1 μM VGVAPG and 1 μM VVGPGA, we saw that only VGVAPG decreased resazurin reduction by 11.05% as compared to the control. Treatment with NAC alone caused an increase in resazurin reduction by 35.64% as compared to the control. However, NAC co-treatment with VGVAPG did not affect resazurin reduction when compared to the control (Fig. 3b).

### Real-Time PCR Analysis of mRNAs

**Fig. 4:**
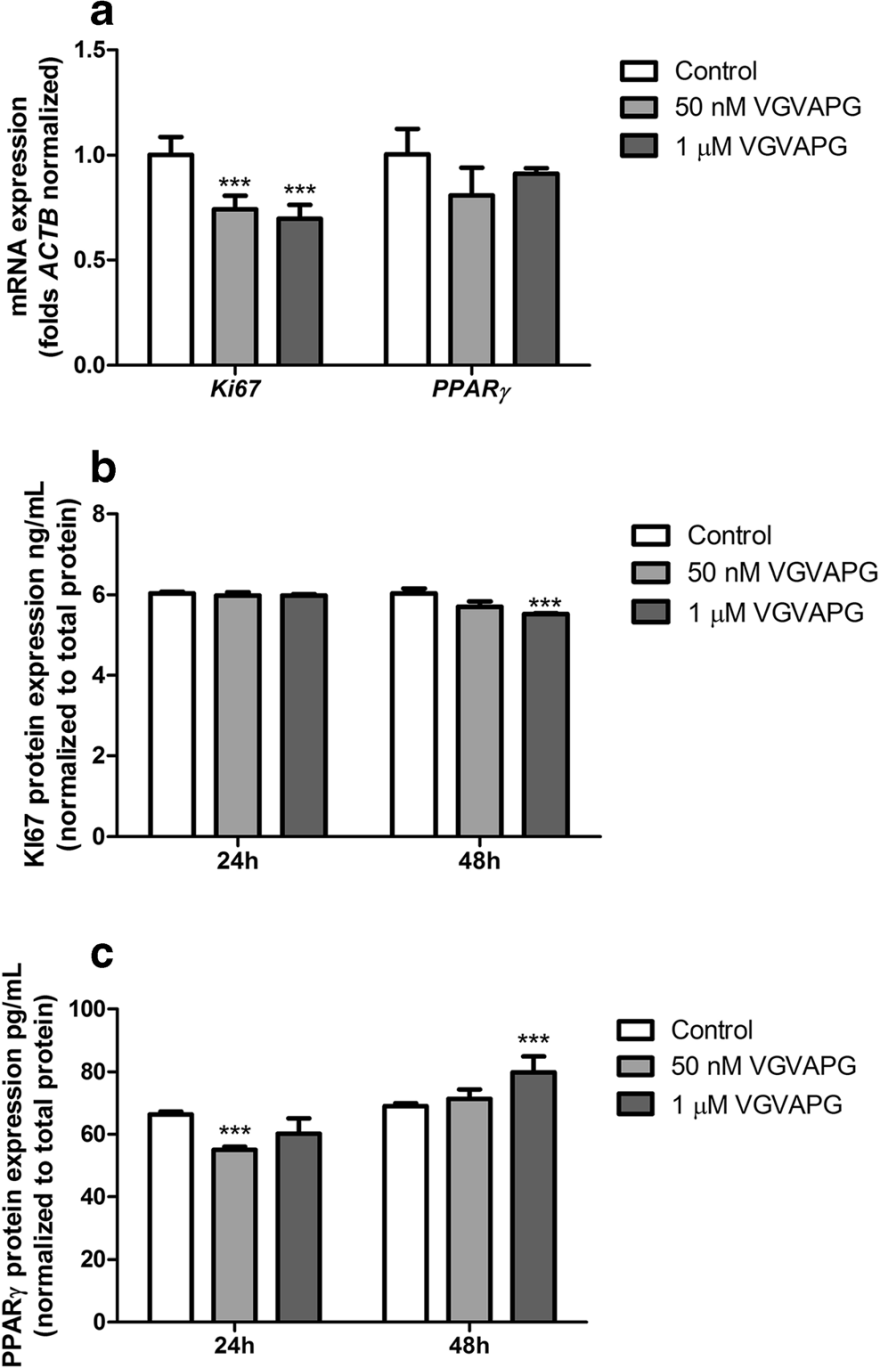
**a** The effect of 50 nM and 1 μM VGVAPG peptide on *Ki67* and *PPARγ* mRNA expression in the SH-SY5Y cell line after 6 h. mRNA expression was normalized to *Actb*. The effect of 50 nM and 1 μM VGVAPG peptide on **b** Ki67 and **c** PPARγ protein expression in the SH-SY5Y cell line after 24 and 48 h. Protein expression was normalized to the total protein level. Data are expressed as mean ± SD of three independent experiments, each of which comprised six replicates per treatment group. *p < 0.05; ***p < 0.001 versus the vehicle control

After 6-h exposure of SH-SY5Y cells to 50 nM and 1 μM VGVAPG peptide, the expression of *KI67* mRNA decreased by 27.77% and 31.50%, respectively, as compared to the control. However, the expression of *PPARγ* mRNA was unaffected by the VGVAPG peptide (Fig. 4a).

### Enzyme-Linked Immunosorbent Assays

#### Ki67 Protein Expression

Exposure of SH-SY5Y cells to 50 nM and 1 μM VGVAPG peptide for 24 h did not change the expression of Ki67 protein; however, after 48-h exposure of SH-SY5Y cells to 1 μM VGVAPG peptide, the expression of the Ki67 protein decreased by 0.51 ng/mL, compared to the control (Fig. 4b).

#### PPARγ Protein Expression

After 24-h exposure of SH-SY5Y cells to 50 nM VGVAPG peptide, the expression of PPARγ protein decreased by 11.33 pg/mL as compared to that with the control. Moreover, 48-h exposure of SH-SY5Y cells to 1 μM VGVAPG peptide increased the expression of PPARγ protein by 10.80 pg/mL as compared to the control (Fig. 4c).

#### GPx Protein Expression

**Fig. 5:**
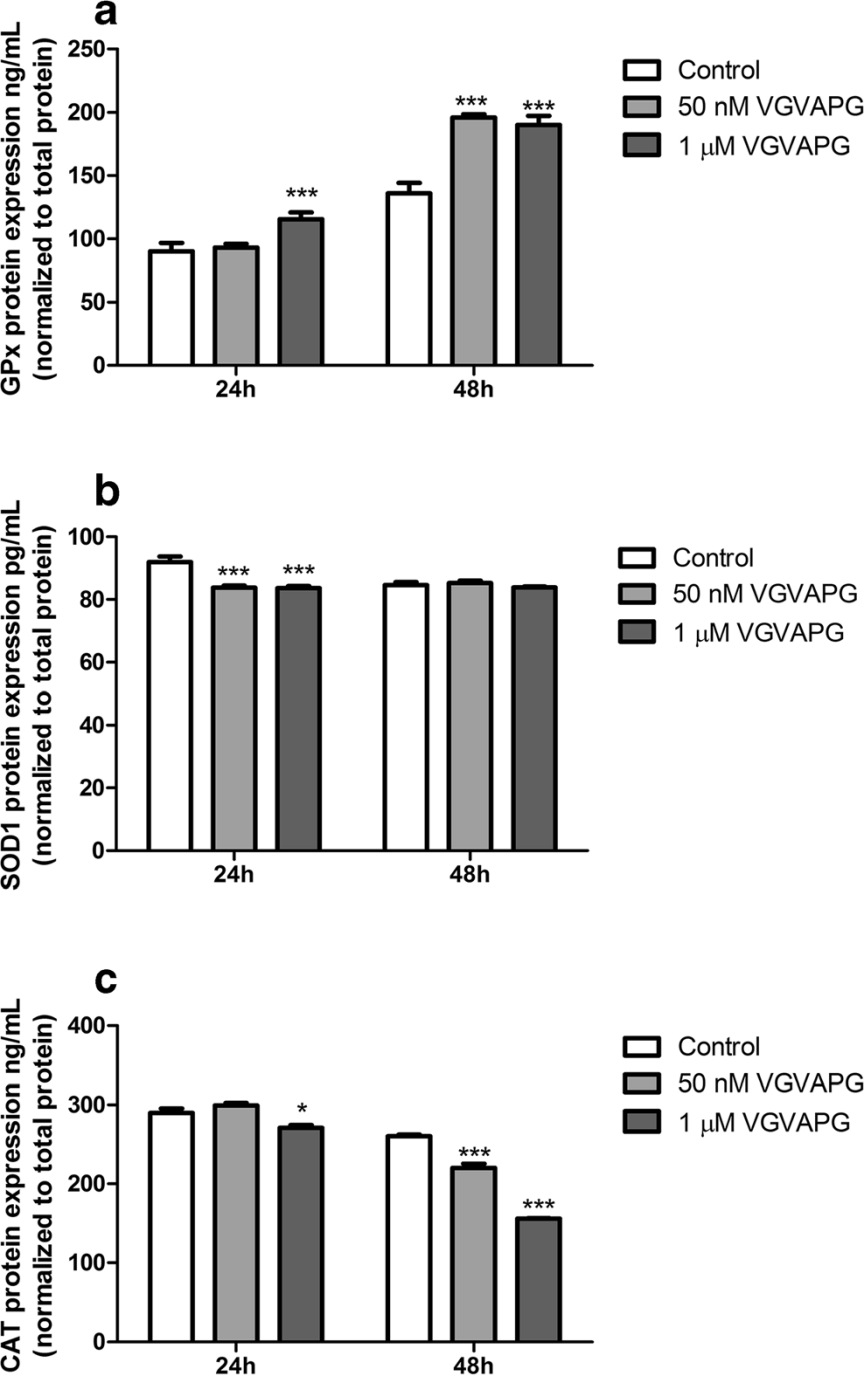
The effect of 50 nM and 1 μM VGVAPG peptide on **a** GPx, **b** SOD1, and **c** CAT protein expression in the SH-SY5Y cell line after 24 and 48 h of exposure. Protein expression was normalized to the total protein level. Data are expressed as mean ± SD of three independent experiments, each of which consisted of six replicates per treatment group. *p < 0.05; ***p < 0.001 versus the vehicle control

After 24-h exposure of SH-SY5Y cells to VGVAPG peptide, the protein expression of GPx in the 1 μM concentration sample increased by 25.90 ng/mL. After 48-h of cell exposure to the VGVAPG peptide, the GPx protein expression increased in both concentrations—50 nM and 1 μM (increase of 60.34 and 54.28 ng/mL, respectively, compared to control; Fig. 5a).

#### SOD1 Protein Expression

Following 24-h exposure to VGVAPG peptide, the protein expression of SOD1 decreased for both the studied peptide concentrations (decreased by 8.15 and 8.29 pg/mL, respectively); however, after 48-h exposure, the protein expression of SOD1 had not changed as compared to the control (Fig. 5b).

#### CAT Protein Expression

Exposure to VGVAPG peptide for 24 h resulted in a decrease of CAT protein expression by 18.95 ng/mL in the sample with a concentration of 1 μM as compared to the control; however, after 48-h exposure, CAT protein expression decreased for both the studied concentrations of 50 nM and 1 μM (decreased by 40.42 and 104.75 ng/mL respectively, compared to control; Fig. 5c).

### Enzyme Activity Assays

#### GPx Activity Assay

**Fig. 6:**
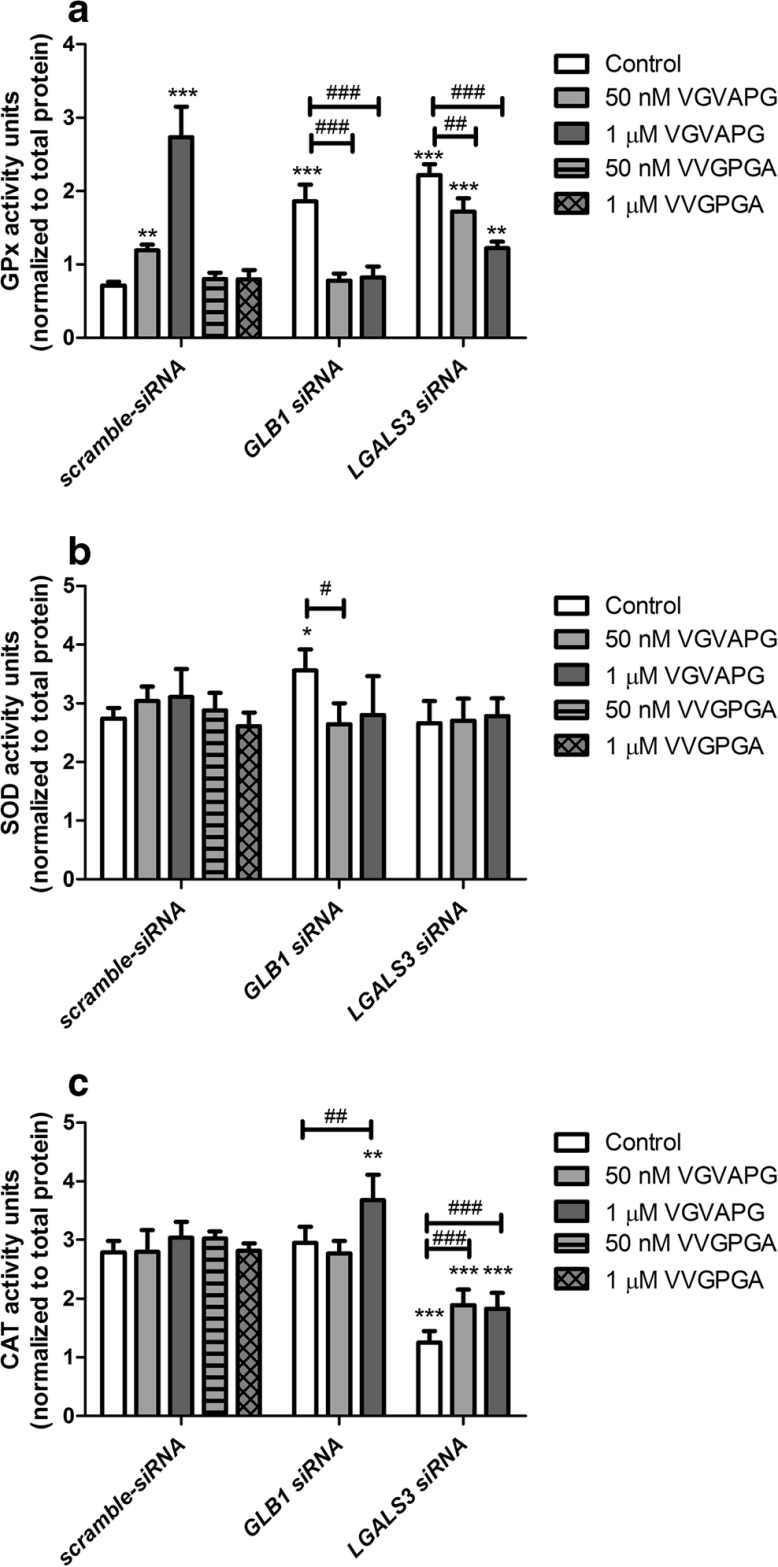
The effect of 50 nM and 1 μM of VGVAPG and VVGPGA peptide, respectively, on **a** GPx, **b** SOD, and **c** CAT activity in the SH-SY5Y cell line after 48 h. The experiments were conducted after application of scrambled siRNA, *GLB1* siRNA, or *LGAL3* siRNA. Protein activity was normalized to the total protein level. Data are expressed as mean ± SD of three independent experiments, each of which consisted of six replicates per treatment group. *p < 0.05; ***p < 0.001 versus the scramble siRNA vehicle control. #*p* < 0.05; ##*p* < 0.01; ###*p* < 0.001 versus the *GLB1* siRNA, or *LGAL3* siRNA vehicle control

Following 48-h exposure to 50 nM and 1 μM of VGVAPG peptide, GPx activity increased by 0.48 and 2.03 units, respectively, as compared to the control. The VVGPGA peptide does not affect GPx activity in SH-SY5Y cells. Silencing of the *GLB1* gene resulted in an increase in GPx activity by 1.15 units as compared to scramble-siRNA-treated cells; however, exposure of cells to 50 nM or 1 μM VGVAPG peptide decreased GPx activity to that of the *GLB1* siRNA control level. Silencing of the *LGALS3* gene induced an increase in GPx activity by 1.51 units as compared to that in scramble-siRNA-treated cells, whereas 50 nM and 1 μM VGVAPG reduced GPx activity by 0.50 and 1.00 units compared to the *LGALS3* siRNA control (Fig. 6a).

#### SOD Activity Assay

After 48-h exposure to 50 nM and 1 μM VGVAPG and 50 nM and 1 μM VVGPGA, none of the studied concentrations were found to affect SOD activity in SH-SY5Y cells. Silencing of the *GLB1* gene induced an increase in SOD activity by 0.81 units compared to scramble-siRNA-treated cells; however, exposure of cells to 50 nM or 1 μM VGVAPG peptide decreased SOD activity to the *GLB1* siRNA control level. Silencing of the *LGALS3* gene does not affect SOD activity in SH-SY5Y cells (Fig. 6b).

#### CAT Activity Assay

After 48-h exposure to 50 nM and 1 μM of VGVAPG and 50 nM and 1 μM VVGPGA, none of the studied concentrations were found to affect CAT activity in SH-SY5Y cells. Silencing of the *GLB1* gene does not affect CAT activity in SH-SY5Y cells; however, exposure of cells to 1 μM VGVAPG peptide induced an increase in CAT activity by 0.73 units compared to the *GLB1* siRNA control level. Silencing of the *LGALS3* gene resulted in a decrease in CAT activity by 1.53 units as compared to the scramble-siRNA-treated cells. After *LGALS3* gene silencing, exposure of cells to 50 nM and 1 μM VGVAPG induced an increase in CAT activity by 0.64 and 0.58 units, respectively, compared to the *LGALS3* siRNA control (Fig. 6c).

## Discussion

This study presents the first data from an investigation of the impact of VGVAPG peptides on SH-SY5Y cells. The experiments revealed that, after 3 and 6 h, VGVAPG peptides at a wide range of concentrations (10 nM to 50 μM) increase ROS production in SH-SY5Y cells, whereas a random peptide sequence (VVGPGA) does not affect ROS production. To date, similar results were obtained by our research group in mouse primary astrocytes. In mouse primary cells, the VGVAPG peptide induced an increase in ROS production in all studied concentrations (100 pM to 100 μM) (Szychowski and Gmiński 2019a). Moreover, it had been described that the κ-elastin and VGVAPG peptide increases ROS production in murine monocytes and human fibroblasts (Robert et al. 1984; Gayral et al. 2014; Scandolera et al. 2015). Scandolera et al. (2015) described that, in an in vitro model of aging, the ROS level in fibroblasts increased, whereas the proliferation potential decreased (Scandolera et al. 2015). Furthermore, during in vitro aging, the proliferatory potential of the VGVAPG peptide was reduced. Due to the key role of ROS in cell signaling, cell death, and aging, we decide to measure the metabolism and proliferation potential of SH-SY5Y cells.

Our data show that, after 24 and 48 h of stimulation of SH-SY5Y cells by the VGVAPG peptide, cell metabolism/proliferation decreased, as measured by the resazurin assay. This decrease in cell metabolism/proliferation was observed in similar concentrations that also caused an increase in ROS production, whereas a random peptide sequence (VVGPGA) did not affect cell metabolism/proliferation. The decrease in the proliferation rate was confirmed by mRNA and protein expression of Ki67 protein, which is an established marker of cell proliferation. To date, it is believed that activation of EBP increases the proliferation of human lymphocytes, fibroblasts, melanoma, astrocytoma, glioma, and endothelial cells (Péterszegi et al. 1996; Tajima et al. 1997; Jung et al. 1998; Hinek et al. 1999; Dutoya et al. 2000; Devy et al. 2010). However, some authors described that, based on the concentration, κ-elastin can stimulate not only proliferation but also cell death (Péterszegi and Robert 1998). Péterszegi and Robert showed that lymphocytes exposed to 1–10 μg/mL of κ-elastin do proliferate; however, when the concentration was greater than 100 μg/mL, cell death was apparent. Interestingly, cells died through both apoptotic and non-apoptotic mechanisms (Péterszegi and Robert 1998). However, in our previous studies, we did not observe an increase in lactate dehydrogenase (LDH) release, which is a marker of necrosis or secondary necrosis (after the disintegration of apoptotic bodies in an in vitro model) in mouse astrocytes stimulated by the VGVAPG peptide (Szychowski et al. 2019b). On the other hand, EDP and/or VGVAPG induce an increase in cell chemotaxis and migration (mainly in cancerous cells) that can lead to decreased cell proliferation without a reduction in cell viability as a result of cell movement (Scandolera et al. 2016). Our previous studies show that the VGVAPG peptide activates and increases PPARγ mRNA and protein expression in mouse astrocytes in vitro (Szychowski and Gmiński 2019b). To date, it is well described that PPARγ stops cell proliferation and promotes cell differentiation (Wada et al. 2006; Stergiopoulos and Politis 2013).

To elucidate the involvement of ROS in decreased cell proliferation, the ROS scavenger NAC was employed. Our experiments show that NAC decreases ROS production that was stimulated by the VGVAPG peptide. Moreover, NAC itself increased SH-SY5Y proliferation when measured by the resazurin reduction assay, and this was confirmed by the ELISA measurement of Ki67 protein. In cells co-treated with NAC and VGVAPG, we did not observe any changes as compared to control cells. Our data suggest that, in SH-SY5Y cells, ROS is a key element to decrease cell proliferation.

To date, it has been well described that the VGVAPG peptide probably acts only through receptors on the cell surface (Senior et al. 1984; Blood et al. 1988; Maurice et al. 2013). Our previous study showed that, in mouse astrocytes, ROS production was dependent on EBP and, after *Glb1* silencing, the VGVAPG peptide did not activate ROS production in mouse astrocytes in vitro (Szychowski and Gmiński 2019a). Therefore, the next step of our study was to investigate the effect of silencing of *GLB1* and *LGALS3* genes in SH-SY5Y cells. Our data show that after stimulation of SH-SY5Y cells by the VGVAPG peptide, GPx activity increased, whereas silencing of the *GLB1* gene prevented this process. Silencing of the *LGALS3* gene partially prevents an increase in GPx activity induced by the VGVAPG peptide. Interestingly, the silencing process of *GLB1* and *LGALS3* itself induces an increase in GPx activity. We did not observe any changes in SOD activity. Similar to previous findings, the silencing of *GLB1* itself induced an increase in SOD activity in the control group. In contrast to protein expression, CAT activity did not change. The silencing of the *LGALS3* gene decreased CAT activity in the group treated with VGVAPG. The addition of a random peptide sequence (VVGPGA) did not affect the activity of any of the studied enzymes. The effects of the *GLB1* and *LGALS3* gene silencing itself on the activity of antioxidant enzymes are probably the result of a role of β-galactosidase and galectin-3 in cells. Based on our data, we believe that both EBP and galectin-3 receptors are involved in the regulation of activity of antioxidant enzymes in SH-SY5Y cells. Moreover, our experiments show that, after 48-h exposure, the VGVAPG peptide increased GPx expression, did not affect SOD1 expression, and decreased CAT protein expression in SH-SY5Y cells.

To date, data on the activity of antioxidant enzymes after treatment with EDPs or VGVAPG are very limited. Gmiński et al. showed that κ-elastin increases the activities of SOD, CAT, and GPx as well as lipid peroxidation in human fibroblasts (Gmiński et al. 1991). On the other hand, the VGVAPG peptide reduced ROS production in neutrophils (Dupont et al. 2013). However, in our opinion, the results obtained by Dupont et al. (2013) were probably an effect of the increased expression of antioxidant enzymes. It is noteworthy that, in our experiments, the highest level of ROS was noted after 6-h exposure to VGVAPG. However, after 24-h cell treatment with 1 μM VGVAPG peptide, the level of ROS was lower than that recorded after 6-h exposure to the VGVAPG peptide. Our results are probably an effect of the increasing expression and/or activity of GPx. To date, it is well described that PPARγ activation decreases ROS production through the involvement of NF-κB and also increases the production of antioxidant enzymes (Lee et al. 2006; Jung et al. 2007). We previously described that the VGVAPG peptide activates the PPARγ receptor in mouse astrocytes in vitro (Szychowski and Gmiński 2019b). A similar phenomenon was observed in SH-SY5Y cells. After 24-h exposure to VGVAPG, the expression of PPARγ protein slightly decreased in SH-SY5Y cells, which is an effect of receptor degradation after activation. However, after 48-h exposure of SH-SY5Y cells to VGVAPG, the PPARγ protein expression increased. Despite the fact that we show that the VGVAPG peptide increased GPx activity, ROS production increased in SH-SY5Y cells at least for short intervals (up to 6 h). Due to the long duration needed to produce antioxidant enzymes, we believe that this phenomenon is the cause of the postponement of ROS reduction that was observed by other authors. Similar to our data, elevated ROS levels and activities of antioxidant enzyme were previously described in C57BL/6 mice and in the SH-SY5Y cell line models of Parkinson’s disease (Cassarino et al. 1997). In our opinion, the primary reason for the rapid increase in ROS level could be excitotoxicity caused by VGVAPG in neuroblastoma cells. To date, it is well described that the N-methyl-D-aspartic acid receptor (NMDAR) is involved in excitotoxicity-induced cell death in human embryonic stem cell–derived neurons, neuroblasts, and neuroblastoma cells (Sun et al. 2010; Gupta et al. 2013; Nato et al. 2015). NMDAR-mediated excitotoxicity is Ca^2+^-dependent and is typical in the nervous system (Dong et al. 2009). Numerous reports have shown that κ-elastin, EDPs, and the VGVAPG peptide activate the influx of Ca^2+^ into cells, and this process requires EBP activation (Jacob et al. 1987; Faury et al. 1998a, b; Maurice et al. 2013). Different authors have suggested that G proteins and c-Src as well as ERK1/2 or MEK1/2 kinases are involved in signal transduction; however, the molecular mechanism mediating the opening of Ca^2+^ channels remains unclear (Mochizuki et al. 2002; Fahem et al. 2008; Maurice et al. 2013). Moreover, it has been shown that EBP is internalized after activation; therefore, we cannot exclude that VGVAPG or EDP can act from within the cell on cell metabolism (Hinek et al. 1995). In our opinion, excitotoxicity mediated through NMDAR and dependent on ROS production is the likely cause of the decrease in the level of the cell proliferation marker Ki67.

## Conclusion

The VGVAPG peptide increases GPx expression and activity in the SH-SY5Y cell line. Silencing of the *GLB1* gene prevents changes in GPx activity. Despite the fact that the VGVAPG peptide increases GPx expression, it increases the ROS level. Moreover, the VGVAPG peptide induced a decrease in SH-SY5Y proliferation that was prevented by the ROS scavenger NAC. ROS production and decreased proliferation of SH-SY5Y cells are the results of excitotoxicity meditated through a close unrecognized molecular pathway. More studies are needed to elucidate the presently unknown mechanism of action of the VGVAPG peptide in the nervous system.

## Abbreviations

CAT: Catalase
DMSO: Dimethyl sulfoxide
EBP: Elastin-binding protein
ECM: Extracellular matrix
EDPs: Elastin-derived peptides
FBS: Fetal bovine serum
gal-3: Galectin-3
GPx: Glutathione peroxidase
PBS: Phosphate-buffered saline
PPARγ: Peroxisome proliferator-activated receptor gamma
ROS: Reactive oxygen species
siRNA: Small interfering RNA
SOD: Superoxide dismutase
VGVAPG: Val-Gly-Val-Ala-Pro-Gly
β-Gal: Beta-galactosidase
